# Pivotal Role of Cardio Thoracic Surgeon in Surgical Management of Metastatic Renal Cell Carcinoma with Supradiaphragmatic Inferior Venecaval Invasion with Long-Term Survival

**DOI:** 10.1186/1749-8090-10-S1-A34

**Published:** 2015-12-16

**Authors:** Bipin Chandra, Surendra K Agarwal, Shantanu Pande, Gauranga Majumdar, Nilesh Srivastav, Anil Mandhani

**Affiliations:** 1 Department Of Cardio Vascular Thoracic Surgery, Sanjay Gandhi Post Graduate Institute Of Medical Sciences, Lucknow, Uttar Pradesh, 226014, India; 2 Department of Urology, Sanjay Gandhi Post Graduate Institute of Medical Sciences, Lucknow, Uttar Pradesh, 226014, India

## Background/Introduction

Inferior venecava (IVC) invasion in renal cell carcinoma (RCC) has an incidence of 4-10% and extension in right atrium is <1%, Lung metastasis is most frequent than at other locations. Supradiaphragmatic IVC thrombus with solitary lung metastasis does not affect the prognosis adversely, provided complete resection is achieved.

## Aims/Objectives

To achieve necessary exposure with complete removal of supradiaphragmatic IVC thrombus, minimise blood loss with support of cardio pulmonary bypass (CPB) with pulmonary metastasectomy in such high risk group of patients.

## Method

We treated 31 patients of RCC with IVC invasion with lung metastasis in one, after categorising under Mayo Classification. Preoperatively metastasis and thrombus extent were determined by computed tomography. Sternotomy or thoracotomy was performed for supradiaphragmatic IVC thrombus, 2 patients were found unfit to undergo surgery on CPB so operated by off-pump intrapericardial IVC clamping under guidance of transoesophageal echocardiography (TOE) and rest were operated on CPB, pulmonary metastasectomy for solitary lung metastasis was performed in one. Patients followed up every 3 months.

## Results

Out of 31 patients, 4 had level 0, 27 had IVC thrombus of these 13 had level I, 4 had level II with solitary pulmonary metastatic lesion in one, 3 had level III and 7 had level IV thrombus and out of these 7 patients, 3 were operated on CPB of which 1 died of disseminated intravascular coagulopathy, 2 patients were operated by off-pump intrapericardial IVC clamping under TOE guidance and 2 patients were inoperable and none had recurrence.

## Discussion/Conclusion

Role of cardio thoracic surgeon is pivotal in achieving a significantly longer overall survival in patients of metastatic RCC with supradiaphragmatic thrombus with solitary lung metastasis, by complete resection of thrombus on or off CPB with metastasectomy and this multidisciplinary approach may benefit patients awaiting fatal outcome of their natural history.

